# SARS-CoV-2 M^pro^ inhibitor identification using a cellular gain-of-signal assay for high-throughput screening

**DOI:** 10.1016/j.slasd.2024.100181

**Published:** 2024-08-22

**Authors:** Renee Delgado, Jyoti Vishwakarma, Seyed Arad Moghadasi, Yuka Otsuka, Justin Shumate, Ashley Cuell, Megan Tansiongco, Christina B. Cooley, Yanjun Chen, Agnieszka Dabrowska, Rahul Basu, Paulina Duhita Anindita, Dahai Luo, Peter I. Dosa, Daniel A. Harki, Thomas Bannister, Louis Scampavia, Timothy P. Spicer, Reuben S. Harris

**Affiliations:** 1 Department of Biochemistry and Structural Biology, University of Texas Health San Antonio, San Antonio, Texas, USA, 78229; 2 Department of Biochemistry, Molecular Biology and Biophysics, University of Minnesota, Minneapolis, Minnesota, USA, 55455; 3 Department of Biochemistry & Molecular Pharmacology, New York University School of Medicine, New York, New York, 10016; 4 Department of Molecular Medicine, The Herbert Wertheim UF Scripps Institute for Biomedical Innovation & Technology, Jupiter, Florida, USA, 33458; 5 Department of Chemistry, Trinity University, San Antonio, Texas, USA, 78212; 6 Lee Kong Chian School of Medicine, Nanyang Technological University, Singapore, Singapore, 308232; 7 Institute of Structural Biology, Nanyang Technological University, Singapore, Singapore, 639798; 8 Department of Medicinal Chemistry, University of Minnesota, Minneapolis, Minnesota, USA, 55455; 9 Howard Hughes Medical Institute, University of Texas Health San Antonio, San Antonio, Texas, USA, 78229

**Keywords:** antiviral drugs, cell-based ultra-high throughput screening (uHTS), protease inhibitors, SARS-CoV-2 main protease (M^pro^/3CL^pro^)

## Abstract

Severe Acute Respiratory Syndrome Coronavirus-2 (SARS-CoV-2, SARS2) is responsible for the COVID-19 pandemic and infections that continue to affect the lives of millions of people worldwide, especially those who are older and/or immunocompromised. The SARS2 main protease enzyme, M^pro^ (also called 3C-like protease, 3CL^pro^), is a *bona fide* drug target as evidenced by potent inhibition with nirmatrelvir and ensitrelvir, the active components of the drugs Paxlovid and Xocova, respectively. However, the existence of nirmatrelvir and ensitrelvir-resistant isolates underscores the need to develop next-generation drugs with different resistance profiles and/or distinct mechanisms of action. Here, we report the results of a high-throughput screen of 649,568 compounds using a cellular gain-of-signal assay. In this assay, M^pro^ inhibits expression of a luciferase reporter, and 8,777 small molecules were considered hits by causing a gain in luciferase activity 3x SD above the sample field activity (6.8% gain-of-signal relative to 100 μM GC376). Single concentration and dose-response gain-of-signal experiments confirmed 3,522/8,762 compounds as candidate inhibitors. In parallel, all initial high-throughput screening hits were tested in a peptide cleavage assay with purified M^pro^ and only 39/8,762 showed inhibition. Importantly, 19/39 compounds (49%) re-tested positive in both SARS2 assays, including two previously reported M^pro^ inhibitors, demonstrating the efficacy of the overall screening strategy. This approach led to the rediscovery of known M^pro^ inhibitors such as calpain inhibitor II, as well as to the discovery of novel compounds that provide chemical information for future drug development efforts.

## Introduction

Severe Acute Respiratory Syndrome Coronavirus-2 (SARS-CoV-2, SARS2) is estimated to have caused over 10 million deaths worldwide from 2019 to present (1–3). Although no longer considered a pandemic by the World Health Organization or the United States Center for Disease Control, SARS2 has become endemic and it continues to infect millions each year, causing cold-like symptoms in the majority, as well as long-term effects collectively termed long-COVID in a minority of patients. Long-COVID pathologies include neurological symptoms (brain fog, changes in taste or smell), respiratory symptoms (heart arrythmia, chest paint, *etc*.), and general symptoms such as fatigue and high fever (1,4,5). SARS2 variants also remain a serious threat to immunocompromised people, including the elderly population, and can trigger pulmonary issues including inflammatory responses that lead to death (6–9).

Vaccines have proven effective in protecting from SARS2 infection and, minimally, lessening its pathogenic effects. However, rapid virus evolution continues to lead to new variants, defined by viral spike protein alterations, that undermine vaccine efficacy and drive periodic vaccine updates from pharmaceutical companies. However, SARS2 infections can also be treated with orally available drugs such as Paxlovid, which includes nirmatrelvir to directly inhibit the activity of the viral main protease enzyme, M^pro^, and ritonavir to inhibit cytochrome P450-mediated metabolism of nirmatrelvir (10–13). Nirmatrelvir blocks M^pro^ from cleaving viral polyprotein substrates into functional units required for viral replication and pathogenesis. Additional M^pro^ inhibitors are in various stages of development ranging from early-stage tool compounds to late-stage clinical trials with ensitrelvir, FB-2001, and PF-07817883 (the active components of Xocova, Bofutrelvir, and Ibuzatrelvir, respectively) (14–19).

Based on clear precedents from prior antiviral drug development campaigns for HIV-1 and hepatitis C virus (HCV) protease enzymes (20–23), it is important to continue to develop and refine M^pro^ inhibitors until potent, long-lasting, orally available compounds are achieved. Next-generation M^pro^ drugs should also be weaned away from ritonavir-dependent regimes, which inhibit cytochrome P450 function and can complicate the use of other medicines. Additional desirable properties for next-generation M^pro^ drugs include broader spectrum activity such that they also inhibit the replication of other coronavirus species including known pathogens such as Middle East Respiratory Syndrome Coronavirus (MERS-CoV), pathogens that do not pose a current threat such as Severe Acute Respiratory Syndrome Coronavirus-1 (SARS1), and related beta-coronaviruses found in bats and many different mammals. Broader spectrum activity against present-day viruses is likely to be a key predictor (though not perfect) of future efficacy against the next pandemic coronavirus (SARS3), which most experts predict will emerge (albeit unclear with respect to timeline) (24–26). Last, but not least, next-generation M^pro^ inhibitors should exhibit different resistance profiles. For instance, the covalent inhibitor nirmatrelvir has a distinct resistance profile from ensitrelvir and FB-2001 (48, 27, 34, 47, 46, 33,35, 43). Here we report the results of an ultra-high-throughput screen (uHTS) of nearly 650,000 compounds using a cell-based assay as a primary screen (33–36). Additionally, as secondary screens, an analogous HCoV-NL63 cell-based assay and a biochemical assay were used to further help delineate candidate inhibitors. The primary screen leverages our original observations that SARS2 M^pro^ overexpression suppresses cellular gene expression (including luciferase reporter gene expression) and that *bona fide* M^pro^ inhibitors recover gene expression in a dose-responsive manner (33–36). Two advantages of using this cellular system as a primary screen are a requirement for cell-permeable molecules and that cytotoxic compounds, which also inhibit gene expression, are unlikely to be identified as positive hits. This cell-based approach led to the rediscovery of known M^pro^ inhibitors, such as calpain inhibitor II, as well as to the discovery of several small molecules that provide chemical information for future drug development efforts.

## Materials and Methods

### Gain-of-Signal Assays for uHTS

First, large batches of 293T cells were pre-transfected (16 μg / 1×10^7^ cells) with pcDNA5/TO-Src-SARS2 M^pro^-Tat-fLuc or Src-NL63 M^pro^-Tat-Luc using Electroporator, ExPERT Stx (SW Version: 4.1.11, MaxCyte, USA). SARS2 and NL63 M^pro^ protein sequences match Genbank accession numbers QII57165.1 and AWK59972.1, respectively. After 4 h incubation at 37°C and 5% CO_2_, cells were harvested and resuspended in Recovery Cell Culture Freezing media (Gibco catalog no. 12648010). Aliquoted cells were frozen slowly at −80°C, then stored in liquid nitrogen until use. The 1,536-well plate format assay begins with thawing batches of pre-transfected cells and dispensing 1250 cells (5 μl) into each well of a 1,536-well plate (Aurora EWB0–42000A). After addition of 50 nl compound or vehicle (for high reference wells, a final concentration of 100 μM GC367 was added), plates were incubated for 48 h at 37°C and 5% CO_2_. Plates were then removed from that environment and incubated at room temperature for 10 min to prevent condensation. Gain-of-signal readouts were initiated by adding 5 μl/well of Bright-Glo reagent (Promega catalog no. E2650), and after an additional 10 min room temperature incubation, firefly luciferase activity was measured using Pherastar instrument (BMG Labtech). The final DMSO concentration per reaction well was 0.75%.

### Z’-Factor Determination

Reproducibility was assessed by calculating a Z-factor (Z’). A Z’-factor of one is considered ideal, and Z’ values measured here (0.47–0.87) are considered robust and significant statistically. Additionally, assay quality can be inferred through a signal-to-noise ratio (S/N) or signal-to-background ratio (S/B). In our efforts to calculate Z’, we used a low reference (24), transfected cells treated with DMSO, and high reference (HR), transfected cells treated with 100 μM GC376, a broad spectrum coronavirus M^pro^ inhibitor (27–33). The following equations were used in which ABS is the absolute value of a number, SD is the standard deviation, and AVR is the average.


$$\text{S}/\text{N}=\frac{\text{AVR}\;\text{of}\;\text{HR}-\text{AVR}\;\text{of}\;\text{LR}}{\text{SD}\;\text{of}\;\text{LR}}$$

$$\text{S}/\text{B}=\frac{\text{AVR}\;\text{of}\;\text{HR}}{\text{AVR}\;\text{of}\;\text{LR}}$$

$${\text{Z}}^{\prime }=1-\frac{3\times \text{SD}\;\text{of}\;\text{LR}\;+3\times \text{SD}\;\text{of}\;\text{HR}}{\text{ABS}(\text{AVR}\;\text{of}\;\text{HR}-\text{AVR}\;\text{of}\;\text{LR})}$$

### Calculating Percent Inhibition in Gain-of-Signal Assay

To determine percent inhibition of M^pro^ at single point concentrations of tool compound GC376, and other relevant chemicals reported in this study, the raw luminescent values (RLU) for each reaction well were used to calculate % inhibition:

$$\%\;\text{Inhibition}=100\times (1-\frac{\text{Test}\;\text{Well}\;\text{RLU}-\text{Median}\;\text{High}\;\text{Control}\;\text{RLU}\left(100\;\mu \text{M}\;\text{GC}376\right)}{\text{Median}\;\text{Low}\;\text{Control}\;\text{RLU}\left(\text{DMSO}\right)-\text{Median}\;\text{High}\;\text{Control}\;\text{RLU}\left(100\;\mu \text{M}\;\text{GC}376\right)}$$

The median low control is derived from transfected cells treated with DMSO, which yields the lowest raw luciferase signal (0% inhibition). The median high control is derived from transfected cells treated with 100 μM GC376, which yields the highest raw luciferase signal (100% inhibition). Candidate inhibitors caused a gain in luciferase signal 3x SD above the sample field activity (6.8% gain-of-signal relative to 100 μM GC376).

### Recombinant Protein Preparation

A pGEX6P-1-SARS2-M^pro^-His6x expression vector, which encodes a glutathione S-transferase (GST)-SARS2 M^pro^-His6x fusion protein was provided by Dr. Shaun Olsen (UT Health San Antonio) (pGEX6P-1 GenBank accession no. QLL57165.1). In this construct, the natural N-terminal cleavage site for M^pro^ is included to facilitate self-cleavage and purification from GST. An P132H derivative (matching Omicron M^pro^) was created by site-directed mutagenesis using primers 5’- ATG-TGC-TAT-GCG-TCA-TAA-TTT-TAC-CAT-TAA-GGG-TAG-3’ and 3’-TAA-TGG-TAA-AAT-TAT-GAC-GCA-TAG-CAC-ATT-GAT-AAA-CGC-5’. After DpnI digestion (New England Biolabs catalog no. 10196884), the PCR product was transformed into chemically competent *E. coli* DH10B cells (Thermo Fisher Scientific catalog no. EC0113). Single colonies were picked, expanded in liquid Luria-Bertani (34) medium supplemented with 100 mg/mL carbenicillin, mini-prepped, and verified by Sanger DNA sequencing.

For protein production, *E. coli* strain BL21(DE3) (New England Biolabs catalog no. C2527H) was transformed with the pGEX6P-1-SARS2-M^pro^-P132H-His6x plasmid, and a single colony was grown overnight to saturation in 50 ml LB medium supplemented with 100 mg/mL carbenicillin (Thermo Fisher Scientific catalog no. J6194903). 5 ml of this primary culture was used to inoculate 1 L of LB broth supplemented with 100 mg/mL of carbenicillin and incubated at 37°C, shaking at 190 rpm, until an optical density (OD) of 0.6 was reached. At this point, M^pro^ expression was induced by adding 0.5 mM IPTG (Thermo Fisher Scientific catalog no. 15529019), and the incubation temperature was lowered to 18°C for an additional 20 h. The cells were collected by centrifugation at 3,000 g, resuspended in 20 mM Tris, pH 8.0, 200 mM NaCl, 5 mM β-mercaptoethanol (Thermo Fisher Scientific catalog no. O33461–100), 5 mM imidazole (Thermo Fisher Scientific catalog no. A1022122), and 5% glycerol (Thermo Fisher Scientific catalog no. A16205AP), and lysed by sonication. M^pro^ was captured from cleared lysate using a nickel-nitrilotriacetic acid gravity flow affinity column (Fisher Scientific catalog no. R90115), washed by a gradient of imidazole, and eluted with 300 mM imidazole. The protein was concentrated using centrifugal filter units (Millipore catalog no. UFC910008) and further purified by size exclusion chromatography (SEC) on a Superdex 200 pg column (Cytvia Life Sciences catalog no. 28989336) operating with 20 mM Tris-HCl, pH 8.0, 150 mM NaCl, 1 mM dithiothreitol (DTT) (Thermo Fisher Scientific catalog no. R0861), and 2% glycerol. The peak fractions of SEC showing single band for M^pro^ in SDS-PAGE were pooled and concentrated to 5 mg/mL as determined by UV absorbance (NanoDrop 8000 spectrophotometer) and, finally, flash frozen in liquid nitrogen for long-term storage at −196°C.

### Biochemical Assay for uHTS

An established biochemical assay (30,35–37) was miniaturized into a 1,536-well plate format with 5 μl/well total reaction volume, which yielded a statistically significant Z’-value of 0.89. First, 2.5 μl of 300 nM SARS2 M^pro^-P132H in reaction buffer [20 mM Tris-HCl, pH 8.0, 150 mM NaCl, 1 mM EDTA, 0.05% Tween20, 0.1 mg/mL bovine serum albumin (BSA), and 1 mM DTT] was dispensed into each well of a 1,536-well plate (Greiner catalog no. 789176-F). Second, 50 nl of each test compound, GC376 (positive control), or DMSO (negative control) was added to each well and reactions were equilibrated for 30 min at RT. Third, 2.5 μl of peptide substrate (DABCYL-KTSAVLQ|SGFRKM-EDANS; UPBio catalog no. V1010–1) in assay buffer (above) was dispensed into each well, and plates were incubated an additional 90 min at RT. The final concentration of M^pro^ was 150 nM, compound was 10.9 μM, and substrate was 5 μM. The final DMSO concentration per reaction well was 0.75%. Fluorescence intensity was measured using a PHERAstar instrument (Ex. 360nm / Em. 460nm filter set), and calculations for inhibition are identical to those used above for cell-based uHTS.

### M^pro^ Inhibition Gain-of-Signal Assay for Purchased Compounds

Candidate M^pro^ inhibitors were purchased from commercial sources for validation studies in 96 well plate format assays (compounds, sources, and catalog numbers are listed in Supplementary Table S1). Well documented compounds were used as positive references throughout these studies including GC376 (28,31–33,38–42), nirmatrelvir (10,11,13,19,25,27,30,32,34,43–49), and boceprevir (29,32,38,39,50,51). These control compounds were purchased from Selleckchem (S0475, S9866, and S3733, respectively). For luciferase-based gain-of-signal assays, 3 × 10^6^ 293T cells were seeded in a 10-cm dish and transfected 24 h later with 2 μg of the pcDNA5/TO-Src-SARS2 M^pro^-Tat-fLuc or Src-NL63 M^pro^-Tat-Luc plasmids (33–36) 4 h post-transfection, the cells were washed once with PBS-EDTA, trypsinized, resuspended, and counted. Cells were diluted in growth medium to yield a suspension of 4 × 10^5^ cells/mL, and 50 μL was plated into each well of a 96-well plate containing 50 μL of growth medium with 2x the desired drug concentration yielding (2 × 10^4^ cells/well with varying compound concentrations). After an additional 44 h incubation, 50 μL of Bright-Glo reagent (Promega catalog no. E2610) was added directly on-top of cell media for a 5-min RT incubation. All reactions were transferred into a white flat bottom 96-well plate (Thermo Fisher Scientific catalog no. 165306) and luminescence was quantified by using a Tecan Spark plate reader (Tecan Life Sciences).

### Calculations to Assess Repurchased Chemicals

To determine the percent inhibition of M^pro^ for a single compound concentration, we used raw luminescent values that have been normalized to DMSO low luminescent control to calculate the percentage of M^pro^ activity as described (33–36):

$$\%\;\text{Mpro}\;\text{activity}=100*\frac{1}{\frac{\text{Test}\;\text{well}\;\text{RLU}}{\text{Mean}\;\text{low}\;\text{control}\;\text{DMSO}\;\text{RLU}}}$$

The mean low control is derived from transfected cells treated with DMSO, which yields the lowest raw luciferase signal (0% inhibition). Second, the normalized percentage of M^pro^ inhibition is calculated by subtracting percent activity (above) from 100:

%Mproinhibition=100-%Mproactivity

Prior to dose response re-testing, all purchased compounds were tested at 20 μM in duplicate and considered inhibitory if 10% of the gain-of-signal activity exhibited by 20 μM of GC376 was reached (*e.g*., equal to or above 9.7% in Figure 4).

### Biochemical M^pro^ Activity Assays for Repurchased Chemicals

The proteolytic activity of SARS2 M^pro^ was analyzed using a quenched fluorescent peptide substrate DABCYL-KTSAVLQ|SGFRKM-EDANS (UPBio catalog no. V1010–1). M^pro^ cleavage between Q and S liberates fluorescence. Cleavage reactions were carried out in 50 μL reactions in Greiner 96-well chimney half-area plates (Greiner catalog no. 675076) with 5 μM substrate, 150 nM M^pro^, 20 mM Tris-HCl, pH 8.0, 150 mM NaCl, 1mM EDTA, 0.05% Tween20, 0.1 mg/mL bovine serum albumin (BSA), 1 mM DTT. For inhibition studies, M^pro^ was incubated at room temperature with various concentrations of chemical (2-fold serial dilution series starting at 100 μM) for 30 m in reaction buffer containing BSA prior to addition of the substrate to initiate the reaction. Fluorescence intensity was measured once per minute using a Tecan Spark 10M plate reader (Ex. 360 nm / Em. 460 nm filter set). The final DMSO concentration per reaction well was 0.75%. Prior to dose response re-testing, all purchased compounds were tested at 20 μM in duplicate and considered inhibitory if 5% of the inhibition of the level of 20 μM of GC376 was reached (*e.g*., equal to or above 5% in Figure 4).

### SARS2 M^pro^ Structures and Molecular Docking

The chemical structures of select compounds were obtained from PubChem, and ChemDraw was used for illustration (Supplementary Table S1). High-resolution x-ray structures of SARS2 M^pro^ with calpain inhibitor II and GC-14 were obtained from the Protein Data Bank (PDB 6XA4 and 8ACL, respectively). PDB 8ACL was also used to for molecular docking studies using Maestro (Schrödinger). The protein was prepared using the Protein Preparation Wizard using default settings with water molecules removed. MWAC-0001776 was sketched in the 2-D sketcher and loaded into the LigPrep tool using an ionization state at pH 7 ± 2 with specified chiralities retained. A docking grid was prepared using the centroid of the workspace ligand, with a hydrogen bond constraint placed at G143. Docking was performed using Glide SP, with constraints. The output of the docked ligand was displayed in the Maestro workspace and used for creating an illustration.

## Results

### Optimization of a Cell-Based Gain-of-Signal Assay for M^pro^ Inhibition

**Figure 1.**
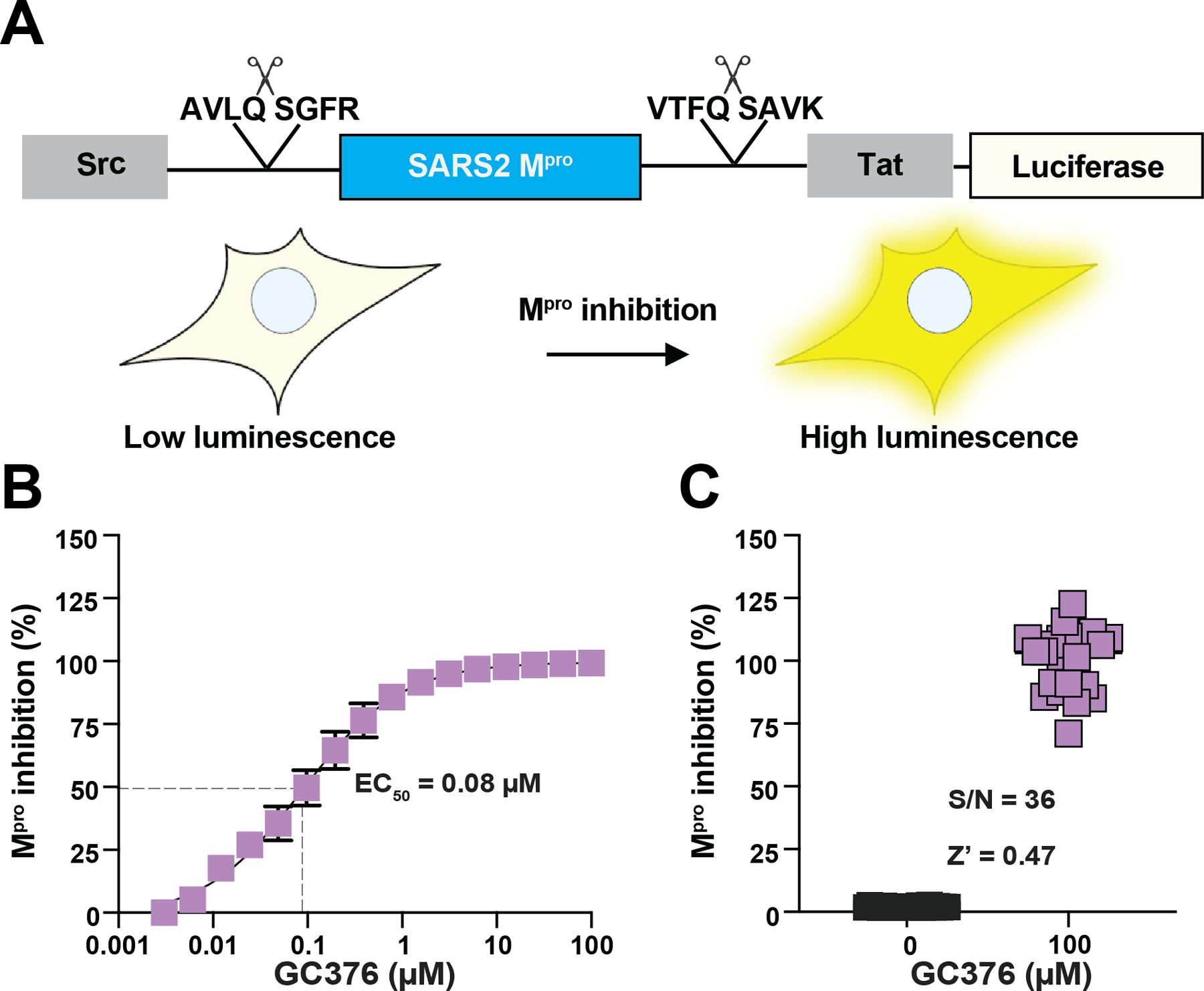
Cell-based gain-of-signal assay for SARS2 M^pro^ inhibition. (**A**) Schematic of cellular gain-of-signal assay for SARS2 M^pro^ inhibition. See text for details. (**B**) Representative dose response with GC376. Each data point is the average of two technical replicates, and the error bars report the difference between each replicate. (**C**) Assay validation in 1536 well format by comparing gain-of-signal assay values for GC-376 and DMSO as a positive and negative controls, respectively (n = 24 for each condition).

We recently reported a cell-based gain-of-signal assay based on the novel observation that wildtype SARS2 M^pro^ suppresses expression of a firefly *luciferase* (34) reporter gene in 293T cells (33–36). In this system, chemical inhibitors of M^pro^ proteolytic activity restore reporter gene expression and luminescent signal in a quantitative and dose-responsive manner (assay schematic in Figure 1A and a dose response of the broad-spectrum coronavirus M^pro^ inhibitor GC376 in Figure 1B). Owing to high sensitivity and a large signal-to-background ratio, the assay was miniaturized to 5 μl total volume and adapted to a 1536-well plate format for uHTS. Using 100 μM of the tool compound GC376 (27–33) as a positive control and DMSO as a negative control, the initial set-up signal-to-background ratio was 36 and the Z’ was 0.47 (Figure 1C).

### Primary uHTS with Gain-of-Signal Assay for M^pro^ Inhibition

Primary uHTS was conducted using the 1536-well format SARS2 M^pro^ gain-of-signal assay and the UF-Scripps Drug Discovery Library (UF-SDDL), which is comprised of 649,568 compounds (52,53). This library is one of the largest in academia, and it is comprised of over 20 commercially sourced compound libraries, supplemented with multiple academically sourced compound series, including small molecules and sub-libraries prepared internally and, therefore, approximately 22,000 compounds in this collection are unique. In its current state, the UF-SDDL has several focused sub-libraries for screening popular drug-discovery target classes (*e.g*., kinases/transferases, GPCRs, ion channels, nuclear receptors, hydrolases, transporters) with diverse chemistries (*e.g*., click-chemistry, PAINS-free collections, Fsp3-enriched, covalent inhibitors, and natural product collections) and desirable physical properties (*e.g*., “rule-of-five”, “rule-of-three”, polar surface area, *etc*.).

**Figure 2.**
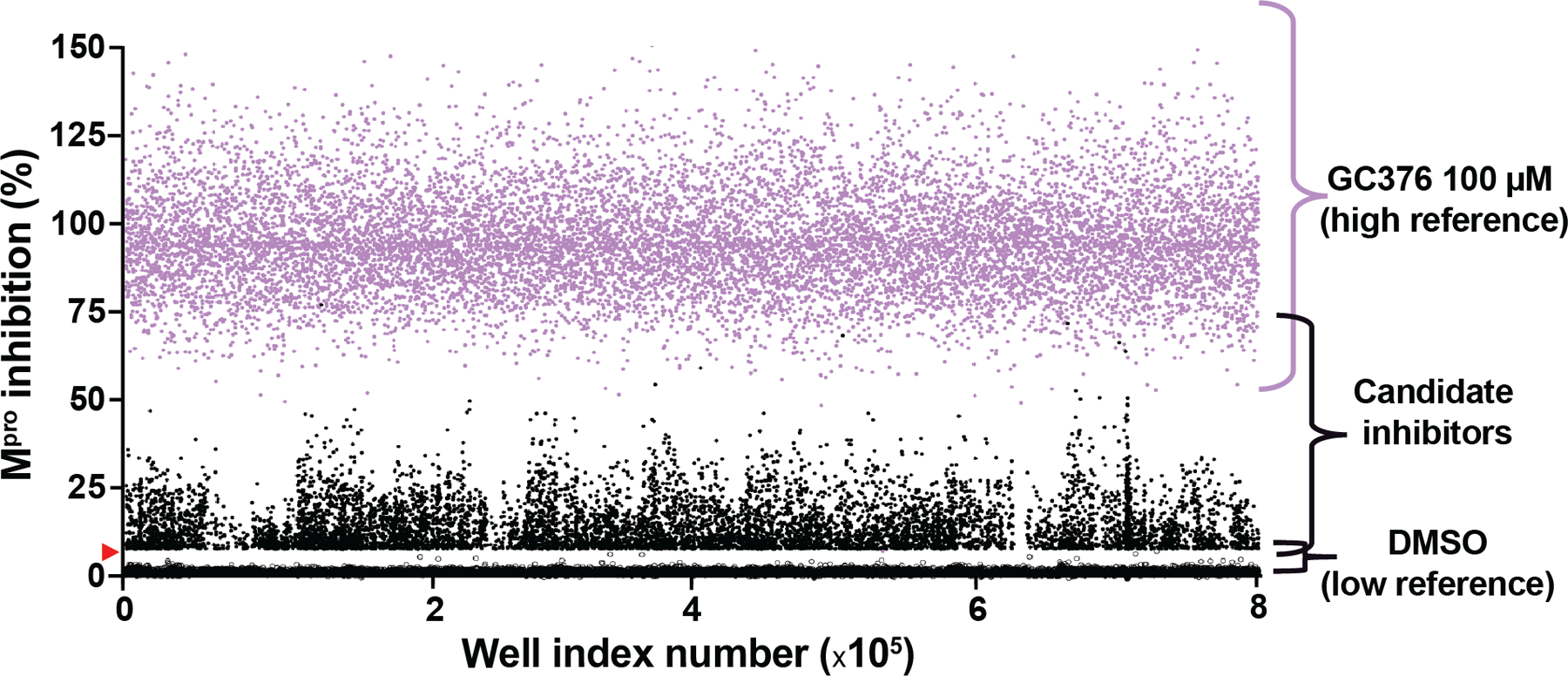
Primary uHTS results for SARS2 M^pro^ inhibition. Data from each 1536 well screening plate are combined and represented as a single dot plot with DMSO values as low controls (open circles) and 100 μM GC376 values as high controls (lavender data points). Candidate inhibitors are represented by black data points with the vast majority falling below the 6.8% gain-of-signal cut-off (not shown to avoid blacking-out DMSO values).

Primary screening was conducted using ~10 μM of each small molecule in single point format, with 24 positive (GC376) and 24 negative (DMSO) wells on every 1536-well plate. The primary screen yielded good statistics, with an average Z’ value of 0.52 +/− 0.08 and a signal-to-background ratio of 32 +/− 7.6 over a total of 522 plates (uHTS composite dot plot in Figure 2). A hit cut-off was established as the average plus 3x SD of sample field activity (6.8% gain-of-signal relative to 100 μM GC376), resulting in a total of 8,777 hits and a final hit rate of 1.35%, in line with results from previous uHTS campaigns (54–56).

### Secondary Screens Using an Orthologous Cell-based Assay with HCoV-NL63 M^pro^ and a Biochemical Peptide-based Cleavage Assay with SARS2 M^pro^

**Figure 3.**
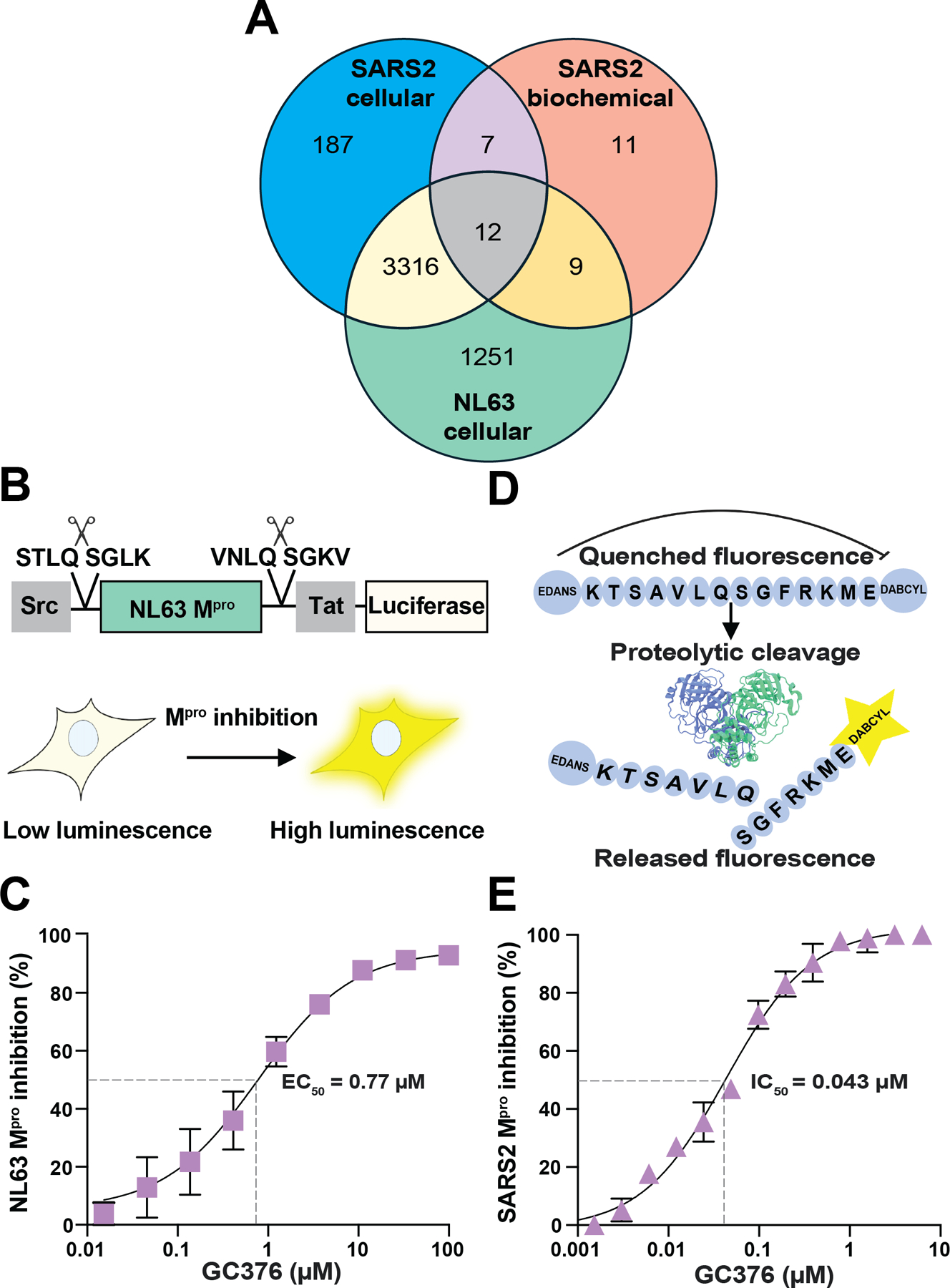
Secondary screens using an orthologous cell-based assay with HCoV-NL63 M^pro^ and a biochemical peptide-based cleavage assay with recombinant SARS2 M^pro^. (**A**) Venn overlap of confirmed positive hits from SARS2 M^pro^ uHTS and secondary screens for inhibition in a cell-based NL63 M^pro^ gain-of signal assay and in a SARS2 M^pro^ biochemical proteolytic cleavage assay. See text for details. (**B**) Schematic of cellular gain-of-signal assay for NL63 M^pro^ inhibition. (**C**) Representative dose response with GC376 in cellular gain-of-signal assay for NL63 M^pro^ inhibition. Each data point is the average of two technical replicates, and the error bars show the difference between each replicate. (**D**) Schematic of the biochemical SARS2 M^pro^ peptide cleavage assay. (**E**) Representative biochemical dose response with GC376. Each data point is the average of two technical replicates, and the error bars show the difference between each replicate.

Primary screen hits were first re-tested in triplicate in 1536-well format with 10.9 μM of each of the 8,762 compounds (15 chemicals from the original 8,777 were unavailable), which resulted in confirmation of 40% of the initial uHTS-implicated small molecules as candidate M^pro^ inhibitors (n = 3,522/8,762; Figure 3A). To further increase the likelihood of discovering direct inhibitors of SARS2 M^pro^ activity, two secondary screens were performed. First, the available candidate SARS2 M^pro^ inhibitors (n = 8,762) were tested on the uHTS platform using an orthologous cell-based assay with a Human Coronavirus NL63 (HCoV-NL63) M^pro^ construct expressed in 293T cells (Src-NL63 M^pro^-Tat-Luc assay schematic in Figure 3B). This construct is identical in amino acid sequence to the SARS2 M^pro^ cell-based construct, apart from the M^pro^ coding region (44% identity), and it was shown previously to suppress luciferase expression to a similar degree in 293T cells (33–36) (representative data with GC376 in Figure 3C). This secondary screen with the HCoV-NL63 construct tested the same 8,762 hits at 10.9 μM and yielded good statistics, with an average Z’ of 0.56 ± 0.05 over 27 plates. Interestingly, many of these compounds inhibited both SARS2 M^pro^ and NL63 M^pro^ (n = 3,328 in Figure 3A). This result was unexpected given 56% divergence between these proteins, and it suggested that these proteases may share at least one cellular target that, when engaged by compound, results in a restoration of luciferase expression. This unexpectedly large group of compounds will be considered in future studies dedicated to identifying the cellular target(s). However, 194 compounds still appeared to uniquely inhibit SARS2 M^pro^ through comparison of the results of these two gain-of-signal cellular assays (Figure 3A).

In parallel, a 1536-well format secondary screen was done with the 8,762 available candidate inhibitors using recombinant SARS2 M^pro^ in an established biochemical assay (30,35–37) (schematic in Figure 3D; see Methods for details). In this assay, limiting amounts of SARS2 M^pro^ (150 nM) are pre-incubated for 30 min with varying concentrations of candidate inhibitor, and then an excess concentration of peptide substrate (5 μM) is added to start the reaction with single hit cleavage kinetics (representative data with GC376 in Figure 3E). Proteolytic cleavage reactions were allowed to proceed at room temperature for 90 min and then data were collected using a plate reader, resulting in similarly high Z’ scores of 0.87 +/− 0.04 over 27 plates.

Interestingly, despite an excellent Z’ scores and a signal-to-background ratio of 6.4 +/− 0.14, only 39 candidate small molecules from the primary cell-based SARS2 uHTS tested positive in this secondary biochemical screen (Venn schematic in Figure 3A). This secondary biochemical screen was stringent in helping to identify direct-binding compounds, as only 19 out of 39 small molecules tested positive both *in vitro* using this assay and in living cells using the SARS2 M^pro^ gain-of-signal assay. Interestingly, 7 of these small molecules appeared specific to SARS2 M^pro^ and the other 12 also showed cross-inhibition of HCoV-NL63 M^pro^ in cells. Both specific and broader-spectrum inhibitors are of interest. Therefore, as an additional test for specificity, these candidate SARS2 M^pro^ inhibitors were tested against purified Zika virus NS2B-NS3 protease in a similar substrate cleavage assay (57). However, 18/19 compounds had no effect on NS2B-NS3 activity, and the outlier (MWAC-0001204) is likely a false positive hit that interferes with the fluorescent readout (Supplementary Table S1).

### Dose Response Studies with Repurchased Compounds

The studies described above were all done with UF Scripps library compounds. To verify these results, all the SARS2 M^pro^ biochemical candidate inhibitors (n = 39), regardless of overlap with the two cell-based assays, together with all the SARS2 M^pro^ gain-of-signal candidate inhibitors (n = 187) were ordered from commercial vendors as powders and solubilized in 100% DMSO for testing. Unfortunately, several of these compounds were unavailable, but a total of 176 small molecules were obtained and tested against SARS2 M^pro^ in our biochemical and cellular gain-of-signal assays (see Methods for details; Supplementary Table S1).

**Figure 4.**
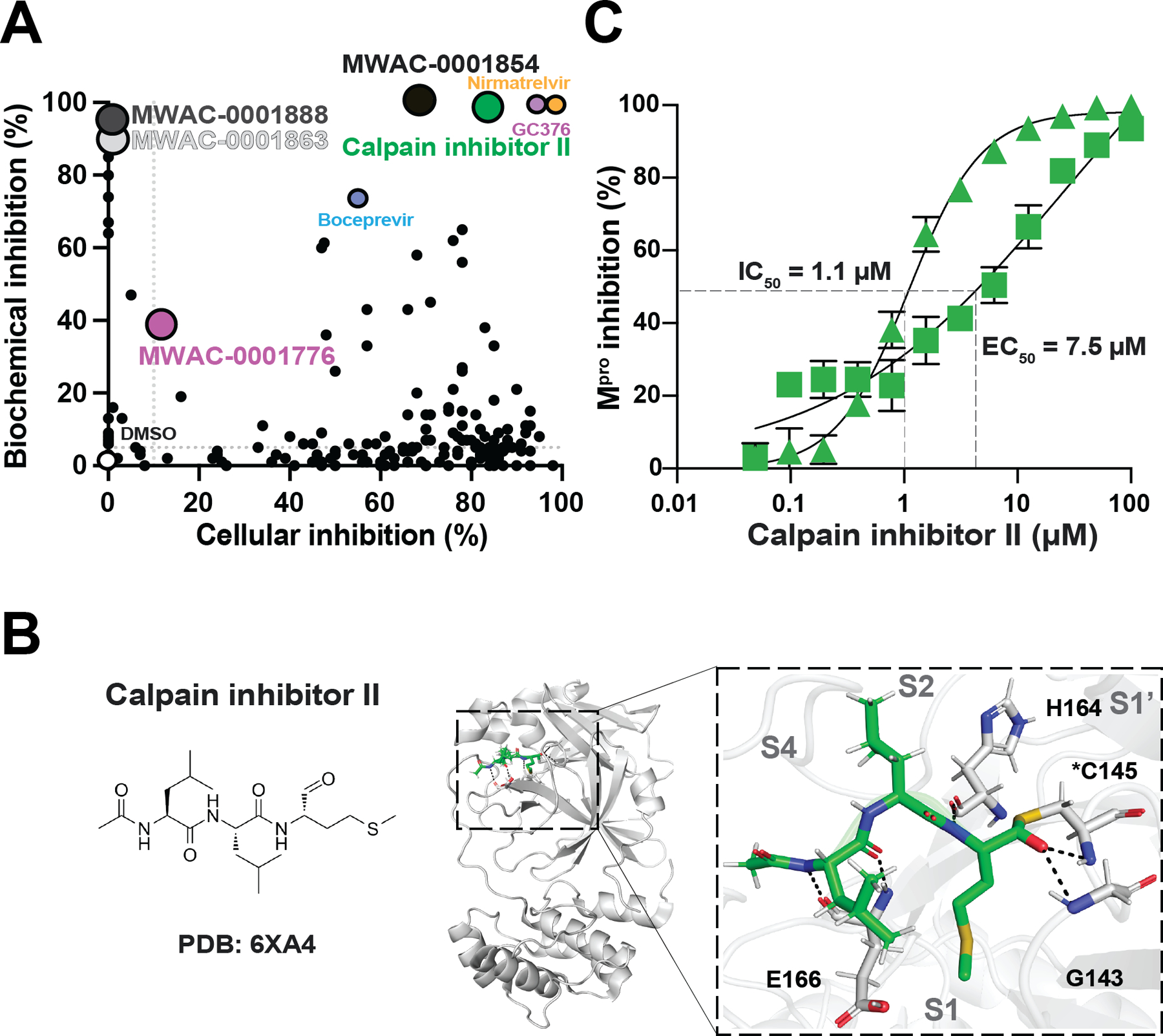
Rediscovery of calpain inhibitor II. **A**) A dot plot comparing cell-based and biochemical SARS2 M^pro^ inhibition results for 20 μM of repurchased compounds. Labels are shown for compounds that were analyzed in detail. Dashed lines indicate significance cut-offs. See text for details. (**B**) Ribbon schematic of the crystal structure of calpain inhibitor II in complex with M^pro^ (PDB 6XA4). The zoom-in (right) shows calpain inhibitor II positioned within the M^pro^ catalytic pocket. Black dashed lines represent hydrogen bonding. (**C**) Representative dose responses with calpain inhibitor II using SARS2 M^pro^ cellular gain-of-signal (square points) and SARS2 M^pro^ biochemical proteolytic cleavage (triangle points) assays. Each data point is the average of two technical replicates, and the error bars show the difference between each replicate.

First, these 176 compounds were tested at a single 20 μM concentration in duplicate and in parallel to various positive controls (Figure 4A and Supplementary Table S1). These experiments yielded a two-dimensional distribution of compound inhibitory activities with the majority showing strong inhibition in the cell-based assay (as identified originally) (Figure 4A). Importantly, half of the compounds tested positive in both assays (87/176; see Methods), alongside positive controls including the strong covalent inhibitor GC376 and the weak covalent inhibitor boceprevir (Figure 4A and Supplementary Table S1). Of note, we re-discovered calpain inhibitor II as a M^pro^ inhibitor with intermediate potency (29, 59, 61). Calpain inhibitor II is a covalent peptidomimetic compound bearing an aldehyde warhead that inhibits calpains and cathepsins, and it was shown to inhibit SARS2 M^pro^ with an IC_50_ of 970 nM in a biochemical assay (29,32,39,58,59). In addition, a SARS2 M^pro^-calpain inhibitor II co-crystal structure revealed that the methionine side chain in the P1 position occupies the S1 subsite (29,32,39,58,59) (Figure 4B). Consistent with these results, our studies with re-purchased compound indicated that calpain inhibitor II has an IC_50_ of 1.1 μM in our biochemical assay and a dose-response EC_50_ of 7.5 μM in our cellular gain-of-signal assay (Figure 4C). This compound also showed no toxicity in 293T cells up to the highest tested concentration (100 μM). These results confirmed that calpain inhibitor II in indeed capable of SARS2 M^pro^ inhibition and further demonstrated the robustness and feasibility of our overall screening approach.

### A New M^pro^ Inhibitor with Similarity to a Reported Small Molecule

**Figure 5.**
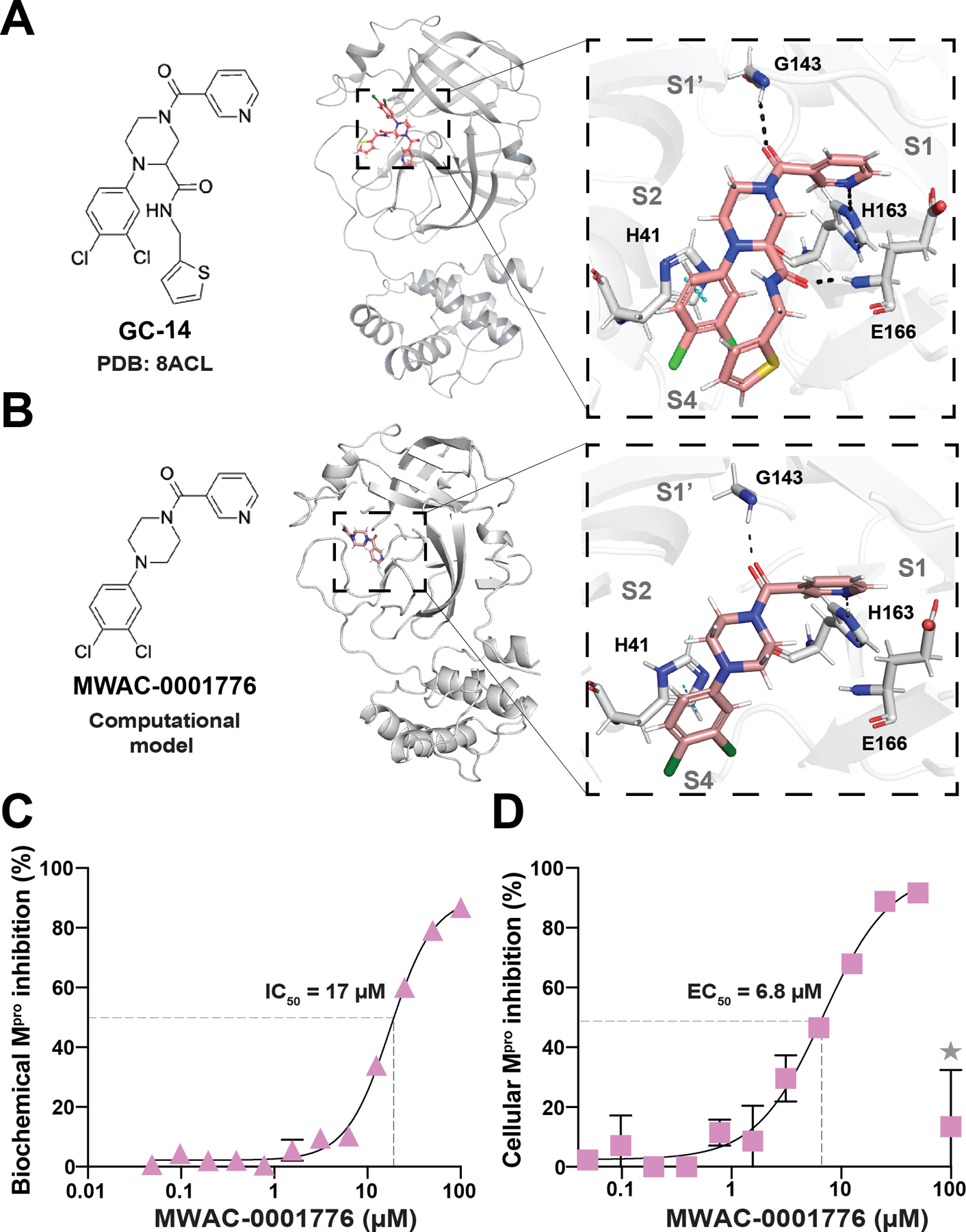
A new M^pro^ inhibitor with similarity to a reported small molecule. (**A**) Ribbon schematic of the crystal structure of GC-14 in complex with M^pro^ (PDB 8ACL). The zoom-in (right) shows GC-14 positioned within the M^pro^ catalytic pocket. Black dashed lines represent hydrogen bonding. Blue dashes represent pi-stacking. (**B**) Chemical structure and computational model of the crystal structure of MWAC-0001776 in complex with M^pro^ model created using Maestro (Schrödinger). The zoom-in (right) shows MWAC-0001776 positioned within the M^pro^ catalytic pocket. Black dashed lines represent hydrogen bonding. Blue dashed represent pi-stacking. (**C**) Representative dose response with MWAC-0001776 using SARS2 M^pro^ biochemical proteolytic cleavage assay. Each data point is the average of two technical replicates, and the error bars show the difference between each replicate. (**D**) Representative dose response with MWAC-0001776 using SARS2 M^pro^ cell-based gain-of-signal assay. Each data point is the average of two technical replicates, and the error bars show the difference between each replicate.

An additional hit from our screening efforts was a non-covalent, disubstituted piperazine, MWAC-0001776, which shares chemical features with a reported compound called GC-14 (60–63) (MWAC-0001776 and GC-14 in Figure 5A–B, respectively). MWAC-0001776 inhibits M^pro^ with an IC_50_ of 17 μM in our biochemical assay and an EC_50_ of 6.8 μM in our gain-of-signal assay (Figure 5C–D). By comparison, GC-14 was reported to exhibit greater potency in a similar biochemical assay (IC_50_ = 0.40 μM) and show activity against SARS2 replication (EC_50_ = 1.1 μM) (60–61). Although GC-14 was not obtained for testing, an obvious difference between MWAC-0001776 and GC-14 is the addition of an amide-linked, 2-aminomethylthiophene on the piperazine of the former compound, which is predicted to occupy the S3/S4 subsite and make polar interactions between the carbonyl of the additional amide bond with the backbone amine of E166 of M^pro^ (Figure 5A). This additional ligand also helps explain why the reported biochemical potency of GC-14 is greater than our observed value for MWAC-0001776. The nicotinyl group that occupies the S1 subsite and the dichlorophenyl that rests in the hydrophobic S2 pocket are identical in the two compounds; this core chemotype may serve as a start point for additional modifications to improve potency.

### Novel Hits Obtained Through uHTS

**Figure 6.**
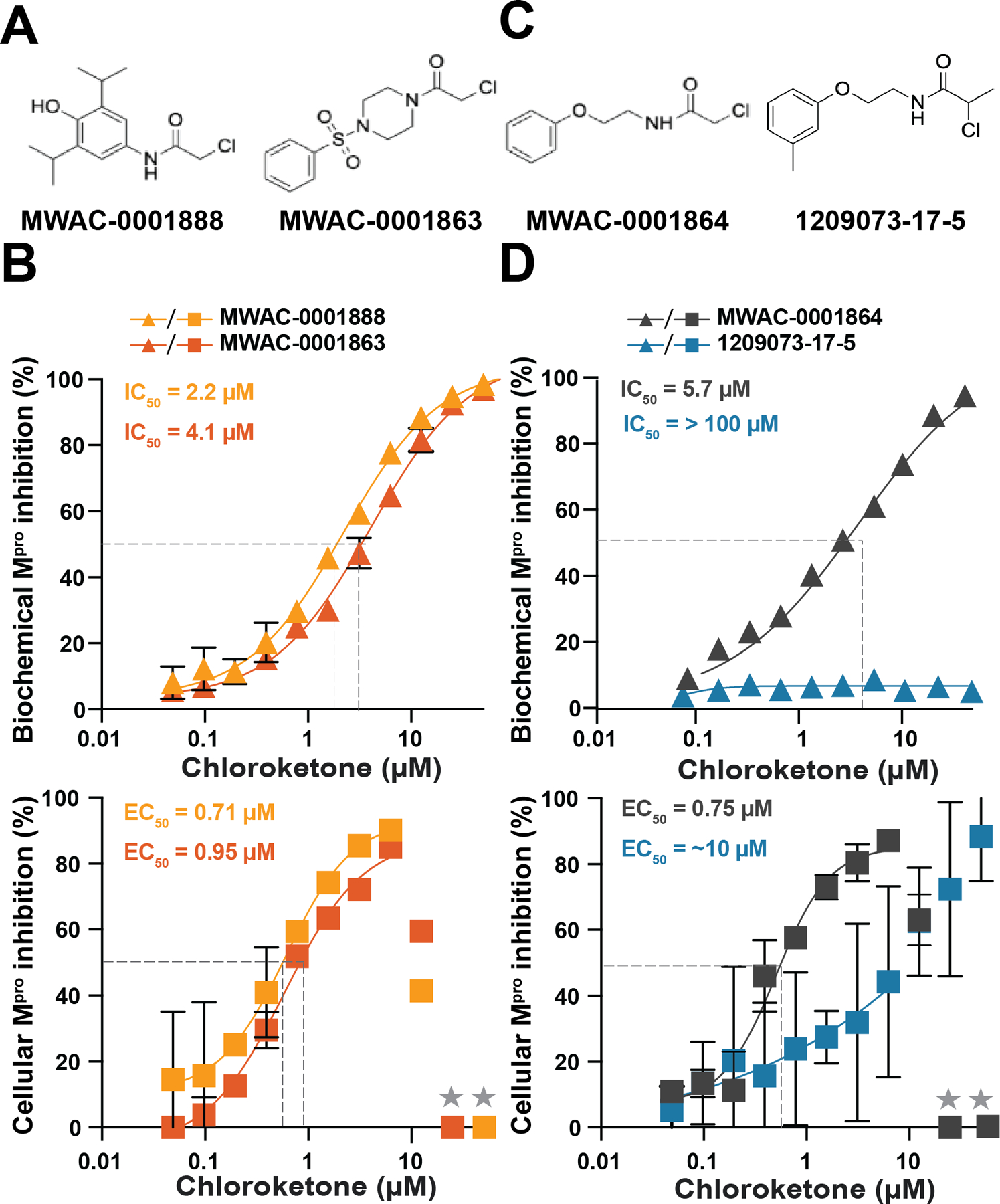
Additional novel hits obtained through uHTS. (**A**) Representative electrophilic α-chloroketone warhead compounds that tested positive in both SARS2 cell-based gain-of-signal and biochemical proteolytic cleavage assays. (**B**) Representative dose response with MWAC-0001863, MWAC-0001854, and MWAC-0001888 using the SARS2 M^pro^ biochemical proteolytic cleavage assay and SARS2 M^pro^ cell-based gain-of-signal assay. Each data point is the average of two technical replicates, and the error bars show the difference between each replicate. (**C**) Representative electrophilic primary (left) and secondary (right) α-chloroketone compounds that were obtained for further characterization. (**D**) Representative dose response with MWAC-000164 and 1209073-17-5 compounds using the SARS2 M^pro^ biochemical proteolytic cleavage assay and SARS2 cell-based gain-of-signal assay. Each data point is the average of two technical replicates, and the error bars show the difference between each replicate.

The largest group of chemically similar compounds among our uHTS hit candidates contained an electrophilic alpha-chloroketone warhead (*e.g*., Figure 6A). These compounds and others with the same electrophilic warhead showed a range of M^pro^ inhibition activity in both our biochemical and cell-based assays (compound information and single concentration results in Supplementary Table S2). Dose response testing was not done for all compounds in this series but select compounds, such as MWAC-0001888 and MWAC-0001863, showed reproducible biochemical IC_50_ and cellular EC_50_ values (Figure 6B).

The enrichment of hits with a shared alpha-chloroketone electrophile suggested that covalent modification in the binding site, specifically with catalytic cysteine C145, likely plays a critical role in M^pro^ inhibition. To test this idea, we obtained a set of commercially available analogs with the alpha-chlorine removed, and all activity in the biochemical assay was abrogated (Supplementary Table S2). These did not warrant testing in cell-based studies.

We next tested a series of 15 commercially available compounds that shared an alpha-chloroketone moiety, and we found that the vast majority of these compounds retained M^pro^ inhibition activity (Supplementary Table S2). A key exception was a modified analog that replaced the primary chloride with a secondary chloride and consequently lost all activity (compare MWAC-0001864 and 1209073-17-5 in Figure 6C–D). To explain this, one would predict a difference in activity due to steric and electronic effects attributable to the addition of the methyl group, which makes the alpha-chloroketone a secondary alkylhalide.

Although the vast majority of the primary alpha-chloroketone-containing compound series showed M^pro^ inhibition, they also caused cytotoxicity at higher concentrations, which might be due to non-specific modification of host proteins and/or affecting cellular redox processes. This is evidenced in dose response curves by overt cell death and extinguished luminescence at higher compound concentrations (*e.g*., higher concentration data points to right of dotted line in Figure 6B). These results may be used in future studies to add specificity through further chemical modifications and/or endow a non-covalent scaffold with irreversible covalent adduction properties of the alpha-chloroketone group.

## Discussion

Coronaviruses have caused three pandemics/endemics in the past 20 years, including SARS1, MERS, and SARS2/COVID-19. However, unlike SARS1 and MERS coronaviruses, which have dissipated naturally or remained restricted geographically, SARS2 has disseminated globally and is likely to continue circulating in humans with the continual emergence of new variants that may render current antiviral medicines less effective. Therefore, it is important to continue to develop and refine M^pro^ inhibitors until potent, long-lasting, orally available compounds are achieved. Here, we report the results of a cell-based ultra-high throughput screen and secondary screens that combined to rediscover known inhibitors and yield new chemical information. Notable small molecules include calpain inhibitor II, as reported (29,32,39,58,59) and MWAC-0001776, which shares core features with a compound called GC-14 (60–63). These two chemotypes are candidates for further development as coronavirus M^pro^ inhibitors.

The largest group of candidate SARS2 M^pro^ inhibitors shared an alpha-chloroketone motif. It is likely that the alpha-chloroketone electrophile inhibits M^pro^ by reacting covalently with the catalytic pocket cysteine, C145. Consistent with this predication, commercially obtained analogs that lacked the alpha-chloroketone group were no longer capable of M^pro^ inhibition.

We recognize that single point, IC_50_ measurements of covalent inhibitors are not generally accepted as rigorous measurements for covalent enzyme inhibition given the time- and concentration-dependent kinetics associated with covalent adduction. Measurements of k_inact_/Ki are generally required during ligand optimization studies. However, given that these hits are still early stage, assays to measure these kinetic parameters will be part of future studies with more potent analogs.

Most M^pro^ inhibitor screens to-date have leveraged biochemical or computational approaches as a first step. The uHTS campaign reported here is the first to our knowledge to use a cell-based gain-of-signal assay for primary HTS. Two advantages of this approach are the immediate identification of candidate small molecules that exert activity in cells and, importantly, are not cytotoxic (at the concentration screened). However, an unexpected drawback of this approach is evidenced by the relatively small number of primary screen hits that were shown to inhibit purified M^pro^ in a subsequent secondary screen (n = 39). Thus, the vast majority of primary screen hits appeared to be causing a gain-of-signal luminescent read-out without directly inhibiting SARS2 M^pro^ inside of cells. The fact that nearly all of these compounds (n = 3328) also caused a gain-of-signal in an orthologous NL-63 M^pro^ cellular assay strongly suggests shared cellular targets. This phenotype may be relevant to the biology of the coronavirus main protease enzyme, and it will be the subject of future mechanistic studies.

## Supplementary Material

Supplementary table S1

Supplementary table S2
